# Direct Renin Inhibition With Aliskiren Compared to Angiotensin-Converting Enzyme Inhibitors and Angiotensin Receptor Blockers in Hypertension: A Systematic Review

**DOI:** 10.7759/cureus.96886

**Published:** 2025-11-15

**Authors:** Abubakar Gapizov, Ahmad Mohammad, Shivam Singla, Bhavna Singla, Sunita Kumawat, Ajay, Zulqurnain Ali

**Affiliations:** 1 Internal Medicine, NewYork-Presbyterian Brooklyn Methodist Hospital, New York, USA; 2 Internal Medicine, Hurley Medical Center, Flint, USA; 3 Internal Medicine, TidalHealth Peninsula Regional, Salisbury, USA; 4 Internal Medicine, Erie County Medical Center Health Campus, Buffalo, USA; 5 Internal Medicine, St. Francis Medical Center, Lynwood, USA; 6 Internal Medicine, Liaquat University of Medical and Health Sciences, Jamshoro, PAK; 7 Internal Medicine, Rawalpindi Medical University, Rawalpindi, PAK

**Keywords:** ace inhibitors, albuminuria, aliskiren, angiotensin receptor blockers, chronic kidney disease, hypertension, renal hemodynamics, renin inhibitors, systematic review, type 2 diabetes

## Abstract

This systematic review evaluated the comparative efficacy of direct renin inhibitors, primarily aliskiren, versus angiotensin-converting enzyme inhibitors (ACEi) and angiotensin receptor blockers (ARBs) in the management of hypertension across varied patient populations. A total of 417 records were screened, with five studies meeting inclusion criteria, including four randomized controlled trials and one large prospective registry. Overall, clinic blood pressure reductions were similar between renin inhibitors and ACEi/ARB therapy. Subgroup analyses revealed nuanced differences: ARBs demonstrated superior effects on reducing urinary angiotensinogen and albuminuria in patients with high-normal albuminuria, while aliskiren provided greater reductions in microalbuminuria and systolic blood pressure when used as add-on therapy in type 2 diabetes with uncontrolled hypertension. In obese hypertensive men, aliskiren uniquely reduced filtration fraction and albuminuria, suggesting possible renal hemodynamic advantages. Real-world registry data further supported the effectiveness and tolerability of aliskiren, with comparable blood pressure reductions and safety outcomes to ACEi/ARB therapy, though limitations inherent to non-randomized designs persist. Risk of bias was judged as low to some concerns across studies, reflecting small sample sizes, limited blinding, and heterogeneous populations. Collectively, current evidence suggests that while renin inhibitors may serve as an alternative for patients intolerant to ACEi/ARB, they do not demonstrate clear superiority, and their role may be more relevant in specific subgroups such as obesity-related hypertension or advanced microalbuminuria. Larger, longer-term trials with hard cardiovascular and renal outcomes are warranted to define their optimal place in therapy.

## Introduction and background

Hypertension remains a leading global health challenge, affecting over one billion individuals worldwide and contributing substantially to cardiovascular morbidity and mortality. Despite advances in pharmacological management, the achievement of optimal blood pressure (BP) control remains inconsistent, particularly in patients with comorbid conditions such as chronic kidney disease (CKD) and type 2 diabetes mellitus (T2DM) [1,2]. The renin-angiotensin-aldosterone system (RAAS) plays a central role in the pathophysiology of hypertension, making it a cornerstone target for therapeutic intervention [3].

Angiotensin-converting enzyme inhibitors (ACEi) and angiotensin receptor blockers (ARBs) have long been the mainstay of RAAS blockade, providing proven benefits in BP reduction, renal protection, and cardiovascular outcomes [4]. However, both classes act downstream within the RAAS cascade and may not achieve complete suppression of renin activity. This limitation has prompted interest in direct renin inhibitors (DRIs), which act at the initial and rate-limiting step of the cascade, potentially offering a more comprehensive blockade of RAAS activity [5]. Aliskiren, the first and only approved oral DRI, has demonstrated antihypertensive efficacy in various patient populations, but its comparative effectiveness relative to ACEi and ARBs remains an area of ongoing investigation [6].

Several randomized controlled trials (RCTs) have directly compared aliskiren with ACEi and ARBs in hypertensive patients with high-risk features, including CKD, T2DM, and microalbuminuria, focusing on clinical outcomes such as BP control and albuminuria reduction [7]. Additionally, real-world data from large registry-based studies have provided complementary insights into the long-term safety and effectiveness of aliskiren in routine clinical practice. While these studies have generated valuable evidence, findings remain heterogeneous, and the relative role of aliskiren compared with established RAAS inhibitors has yet to be fully clarified [8].

The aim of this systematic review is to evaluate the comparative effectiveness of direct renin inhibition versus ACEi and ARB therapy in patients with hypertension. By synthesizing evidence from RCTs and real-world studies, we seek to provide a comprehensive assessment of clinical, renal, and hemodynamic outcomes, thereby informing clinicians and policymakers on the role of renin inhibitors in contemporary hypertension management.

## Review

Materials and methods

Search Strategy

This systematic review was conducted in accordance with the Preferred Reporting Items for Systematic Reviews and Meta-Analyses (PRISMA) guidelines [9]. A comprehensive literature search was performed using PubMed/MEDLINE, Embase, and the Cochrane Central Register of Controlled Trials (CENTRAL) from inception to January 2025. The search strategy combined Medical Subject Headings (MeSH) and free-text terms: “renin inhibitors”, “direct renin blockade”, “aliskiren”, “angiotensin-converting enzyme inhibitors”, “ACE inhibitors”, “angiotensin receptor blockers”, “ARB”, and “hypertension”. The PubMed search string was constructed as follows: (“Renin”[MeSH Terms] OR “Renin Inhibitors”[MeSH Terms] OR “Direct Renin Inhibition” OR “Aliskiren” OR “Direct Renin Blockade”) AND (“Angiotensin-Converting Enzyme Inhibitors”[MeSH Terms] OR “ACE Inhibitors” OR “Angiotensin II Type 1 Receptor Blockers”[MeSH Terms] OR “ARBs” OR “Losartan” OR “Ramipril”) AND (“Hypertension”[MeSH Terms] OR “High Blood Pressure” OR “Essential Hypertension”) AND (“Randomized Controlled Trial”[Publication Type] OR “Clinical Trial”[Publication Type] OR “Observational Study” OR “Prospective Study”). Boolean operators (AND, OR) were used to ensure both sensitivity and specificity, and equivalent Emtree terms were applied for the Embase search (e.g., ‘renin inhibitor’/exp, ‘angiotensin converting enzyme inhibitor’/exp, ‘angiotensin receptor antagonist’/exp, ‘hypertension’/exp). Filters for RCTs, clinical trials, and observational studies were applied. The time frame covered all available studies up to January 2025, with included trials ranging from 2014 to 2017, consistent with the publication dates of the selected studies. Reference lists of included articles were also screened manually to identify additional eligible studies.

Eligibility Criteria (PICO Framework)

Eligibility for inclusion in this systematic review was defined using the PICO framework [10], which stands for Population, Intervention, Comparator, and Outcomes. The population (P) consisted of adult patients aged 18 years or older with hypertension, including those with comorbid conditions such as T2DM, CKD, or obesity. The intervention (I) of interest was treatment with a direct renin inhibitor, specifically aliskiren. The comparators (C) were ACEi or ARBs, either as monotherapy or as part of combination therapy. The outcomes (O) assessed included primary endpoints such as reductions in BP and albuminuria, while secondary endpoints encompassed renal hemodynamic changes, biomarker modulation (such as urinary angiotensinogen excretion), cardiovascular outcomes, and safety or tolerability. Both RCTs and real-world observational studies that directly compared renin inhibitors with ACEi or ARB therapy were considered eligible. Studies that did not report comparative outcomes, along with non-human studies, reviews, editorials, and case reports, were excluded from this review.

Study Selection

Titles and abstracts identified through the search were screened independently by two reviewers. Full-text articles were retrieved for all studies meeting preliminary criteria, and final inclusion was based on consensus following detailed review. Disagreements were resolved through discussion. The selection process was documented using a PRISMA flow diagram, illustrating the number of studies identified, screened, excluded, and included.

Data Extraction

A structured data extraction sheet was used to collect information from each included study. Extracted variables included study design, sample size, patient characteristics, intervention and comparator regimens, duration of follow-up, and reported outcomes. Where available, effect estimates (mean changes, percentages, confidence intervals, and p-values) were recorded. Extraction was performed by one reviewer and cross-checked by a second to ensure accuracy.

Risk-of-Bias Assessment

The methodological quality of RCTs was assessed using the Cochrane Risk of Bias 2 (RoB 2) tool [11], evaluating domains such as randomization, blinding, missing data, outcome measurement, and selective reporting. The observational registry study was appraised using the Risk Of Bias In Non-randomized Studies of Interventions (ROBINS-I) tool [12], focusing on confounding, selection, measurement, and reporting bias. Each study was rated as “low concern” or “some concerns” overall.

Data Synthesis

Due to heterogeneity in study designs, populations, and outcome measures, meta-analysis was not feasible. Instead, a qualitative narrative synthesis was performed, comparing trial outcomes across clinical settings and highlighting subgroup effects and mechanistic differences. Real-world evidence was discussed separately to provide context on safety and long-term effectiveness.

Results

Study Selection Process

**Figure 1:**
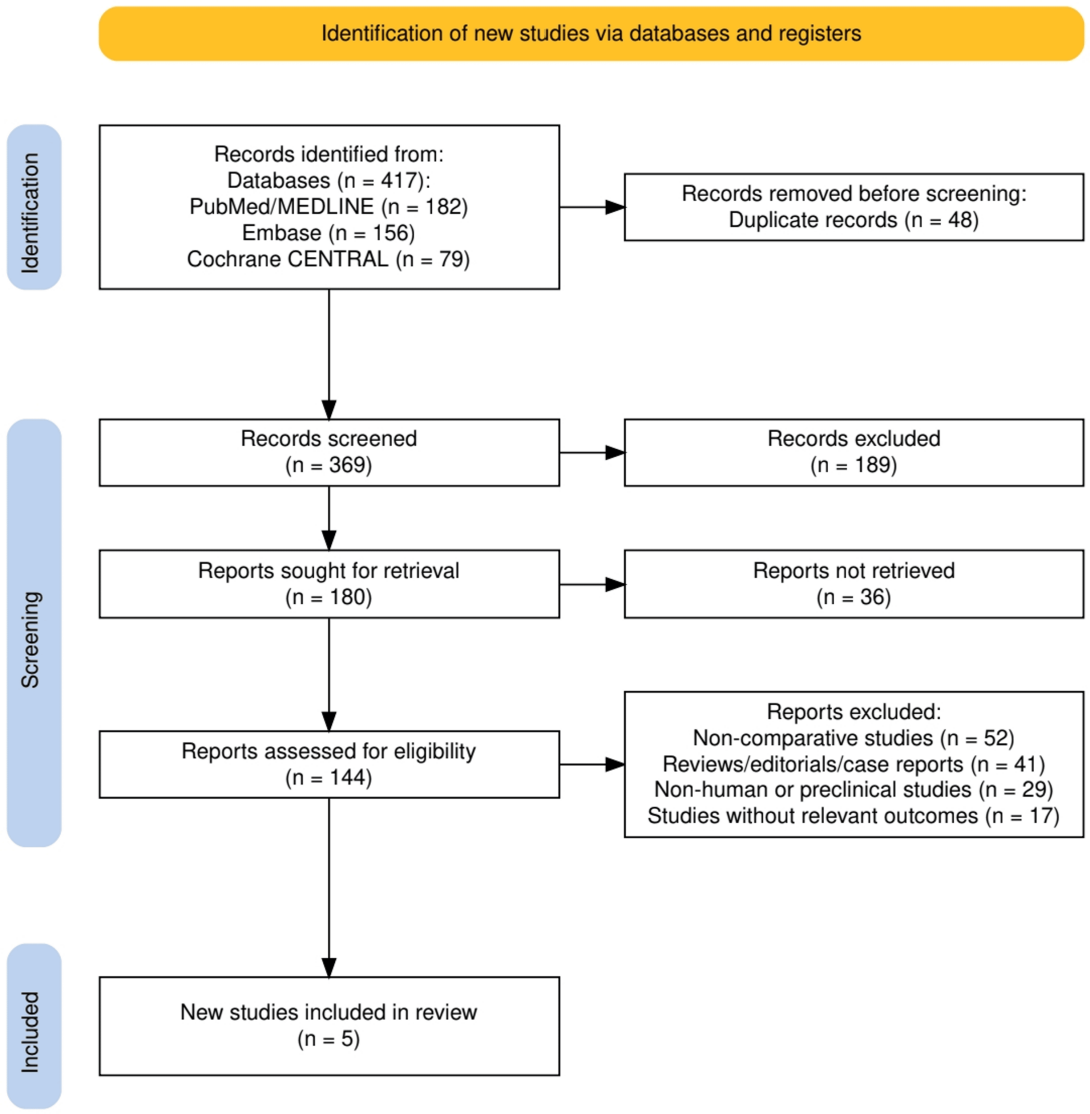
PRISMA flow chart representing the study selection process. PRISMA, Preferred Reporting Items for Systematic Reviews and Meta-Analyses

A total of 417 records were identified through database searching, including 182 from PubMed/MEDLINE, 156 from Embase, and 79 from Cochrane CENTRAL. After removal of 48 duplicates, 369 records underwent title and abstract screening, of which 189 were excluded for not meeting eligibility criteria. The remaining 180 reports were sought for full-text retrieval, with 36 not retrievable. A total of 144 full-text articles were assessed for eligibility, leading to the exclusion of 52 non-comparative studies, 41 reviews/editorials/case reports, 29 non-human or preclinical studies, and 17 studies without relevant outcomes. Ultimately, five studies met the inclusion criteria and were incorporated into the systematic review. The detailed screening and selection process is illustrated in Figure 1.

Characteristics of the Selected Studies

The five included studies comprised four RCTs and one large prospective registry, encompassing diverse hypertensive populations such as those with CKD, T2DM, obesity, and uncontrolled hypertension. As shown in Table 1, all studies evaluated aliskiren against either ACEi or ARBs, with follow-up periods ranging from 24 weeks to two years. Across trials, clinic BP reductions were comparable between treatment groups, though some differences emerged in secondary outcomes. ARBs showed stronger reductions in urinary angiotensinogen and albuminuria among patients with high-normal albuminuria, while aliskiren demonstrated greater reductions in microalbuminuria and systolic BP when used as add-on therapy. In obese hypertensive men, aliskiren uniquely lowered filtration fraction and reduced albuminuria beyond ACE inhibition, suggesting potential renal hemodynamic benefits. Real-world evidence from the registry confirmed effective BP control and tolerability of aliskiren comparable to ACE/ARB therapy, though without differences in major cardiovascular outcomes. Collectively, the studies provide complementary insights into the relative efficacy and safety of direct renin inhibition versus established RAAS blockade across varied clinical settings.

**Table 1:** Summary of randomized controlled trials and real-world evidence comparing renin inhibitors with ACEi and ARBs in hypertension. ACEi, angiotensin-converting enzyme inhibitors; ACR, albumin-to-creatinine ratio; ARB, angiotensin receptor blocker; BP, blood pressure; CKD, chronic kidney disease; DRI, direct renin inhibitor; GFR, glomerular filtration rate; MI, myocardial infarction; RAAS, renin–angiotensin system; RAAS, renin–angiotensin–aldosterone system; T2DM, type 2 diabetes mellitus

Author (Year)	Study Design	Population (N, Characteristics)	Intervention (Renin Inhibitor)	Comparator (ACE/ARB)	Outcomes and Key Findings
Uneda et al. (2016) [13]	RCT	36 hypertensive CKD patients (18 DRI, 18 ARB)	Aliskiren	ARB (unspecified, Ang II type 1 receptor blocker)	After 24 weeks, clinic BP reduction was similar between groups. Ambulatory BP: daytime and nighttime systolic BP A/B ratios were higher in ARB group. Ankle-brachial index was higher with aliskiren. Conclusion: Aliskiren not superior to ARB in lowering ambulatory BP, though clinic BP effects were comparable.
Uzu et al. (2016) [14]	RCT	237 hypertensive type 2 diabetic patients with high-normal albuminuria (10–30 mg/g ACR) or microalbuminuria (30–300 mg/g ACR); 225 completed	Aliskiren (DRI)	Any ARB	After treatment, BP control was similar between groups. Changes in albumin-to-creatinine ratio were comparable (-5.5% DRI vs -6.7% ARB). ARB significantly reduced urinary angiotensinogen, while DRI did not. In subgroup analysis, ARB reduced albuminuria in high-normal patients, but DRI did not. Conclusion: Aliskiren not superior to ARBs for albuminuria reduction or renal protection.
Imbalzano et al. (2015) [15]	RCT (open-label, active comparator)	126 hypertensive type 2 diabetic patients with uncontrolled hypertension and microalbuminuria despite conventional therapy	Aliskiren added to conventional therapy	Ramipril or losartan added to conventional therapy	After 24 weeks, both groups had significant BP reductions, but systolic BP decreased more with aliskiren (-11.37% vs -8.47%, p<0.001). Microalbuminuria decreased significantly in both groups, but reduction was greater with aliskiren (-67.6% vs -49.1%, p<0.001). Conclusion: aliskiren add-on therapy showed superior BP and albuminuria reduction compared with ACEi/ARB add-on therapy.
Kwakernaak et al. (2017) [16]	RCT (double-blind, cross-over)	15 men with weight excess and hypertension	Aliskiren 300 mg/day	Ramipril 10 mg/day (ACEi, positive control)	GFR was unaffected by either therapy. Effective renal plasma flow increased with both aliskiren and ramipril. Filtration fraction was reduced by aliskiren only. Mean arterial pressure decreased with both treatments. RAAS activity was suppressed by both, while albuminuria reduction was observed only with aliskiren. Conclusion: both DRI and ACEi improved renal/systemic hemodynamics and reduced RAAS activity; aliskiren had additional benefit on albuminuria and filtration fraction.
Zeymer et al. (2016) [17]	Prospective non-interventional registry (3A Registry)	8,723 hypertensive patients in routine clinical practice, Germany; patients on aliskiren had more comorbidities and higher baseline BP	Aliskiren (alone or with other antihypertensives)	ACE inhibitors, ARBs, or non-RAS drugs	At 2 years, mean BP reduction was similar across groups (~-20.5/-9.9 mmHg). Rates of death (2.3%), MI (0.5%), stroke (0.6%), hospitalization (2.9%), and overall adverse events (5.5%) did not differ significantly between groups. Aliskiren was effective and well tolerated under real-world conditions. Conclusion: aliskiren provided BP reduction comparable to ACEi/ARB in clinical practice, without major safety concerns, though study limitations prevent firm conclusions on dual RAS blockade.

Risk-of-Bias Assessment

As summarized in Table 2, the risk of bias varied across the included studies. The randomized trials generally showed low concern in outcome measurement, as BP and laboratory parameters were objective endpoints, but most raised some concerns due to limited reporting of randomization, unclear blinding, and potential selective reporting. One open-label trial was particularly vulnerable to performance bias, while the cross-over design of another trial introduced a possible carryover effect despite adequate blinding. The large real-world registry was appraised using the ROBINS-I tool and demonstrated some concerns across several domains, primarily due to treatment-by-indication bias, baseline differences, and variability in adherence typical of observational designs. Overall, while the evidence base is strengthened by multiple RCTs, the predominance of small sample sizes and methodological limitations indicate that findings should be interpreted with caution.

**Table 2:** Risk-of-bias assessment of included RCTs and registry study BP, blood pressure; CKD, chronic kidney disease; RCT, randomized controlled trial; RoB 2, Risk of Bias 2 tool; ROBINS-I, Risk of Bias in Non-randomized Studies of Interventions; T2DM, type 2 diabetes mellitus

Study (Year)	Design	Tool Used	Randomization/Allocation	Blinding and Deviations	Missing Data	Outcome Measurement	Reporting	Overall Risk of Bias	Notes
Uneda et al. (2016) [13]	RCT (parallel)	Cochrane RoB 2	Some concerns (small sample, method not fully described)	Some concerns (blinding unclear)	Low concern	Low concern (BP objective)	Some concerns	Some concerns	Small CKD trial; clinic BP similar, ambulatory BP favored ARB.
Uzu et al. (2016) [14]	RCT (multicenter)	Cochrane RoB 2	Some concerns (randomization details limited)	Some concerns (blinding unclear)	Low concern	Low concern (albuminuria and BP objective)	Some concerns	Some concerns	Large T2DM RCT; ARB reduced angiotensinogen more than aliskiren.
Imbalzano et al. (2015) [15]	RCT (open-label)	Cochrane RoB 2	Some concerns (method not detailed)	Some concerns (open-label design)	Low concern	Low concern (BP and labs objective)	Some concerns	Some concerns	Aliskiren superior for BP and albuminuria, but open-label risk of bias.
Kwakernaak et al. (2017) [16]	RCT (double-blind, cross-over)	Cochrane RoB 2	Low concern	Low concern (blinding ensured)	Low concern	Low concern (hemodynamic/lab objective)	Some concerns (carryover risk)	Low concern	Small mechanistic study; aliskiren reduced albuminuria, ramipril did not.
Zeymer et al. (2016) [17]	Prospective registry	ROBINS-I	Some concerns (treatment by indication, baseline differences)	Some concerns (real-world adherence variability)	Some concerns	Low concern (BP and events objective)	Some concerns	Some concerns	Large registry; aliskiren comparable to ACEi/ARB in real-world practice.

Discussion

This systematic review demonstrates that renin inhibitors, particularly aliskiren, achieve BP reductions broadly comparable to ACEi and ARBs. Across RCTs, clinic systolic BP reductions were consistent between groups, with some trials reporting slightly greater mean reductions in the aliskiren arm (e.g., Imbalzano et al. [15]: −11.4% vs. −8.5% systolic BP). Albuminuria outcomes were mixed: ARBs provided superior reductions in high-normal albuminuria in the study by Uzu et al. [14], whereas aliskiren achieved greater relative reductions in microalbuminuria (−67.6% vs. −49.1%) in the study by Imbalzano et al. [15]. In mechanistic studies, aliskiren uniquely lowered filtration fraction and reduced albuminuria in obese hypertensives [16], highlighting potential subgroup-specific benefits. Real-world registry data (N=8,723 [17]) confirmed effective BP lowering (−20.5/−9.9 mmHg) with aliskiren, comparable to ACEi/ARBs, supporting its clinical utility but with limited causal inference.

RAAS blockade remains the cornerstone of hypertension management, with ACEi and ARBs recommended as first-line agents by current international guidelines [18]. The findings of this review reinforce that direct renin inhibition offers no consistent superiority over ACEi/ARB therapy in terms of BP or renal outcomes. Importantly, large landmark studies were terminated early due to adverse safety signals (hyperkalemia, hypotension, renal dysfunction) when aliskiren was combined with ACEi or ARB, tempering enthusiasm for dual RAAS blockade [19]. Nevertheless, smaller RCTs included here suggest comparable efficacy of aliskiren monotherapy, particularly in high-risk populations such as those with T2DM or CKD. Observational data from the 3A Registry provide additional reassurance about long-term safety and tolerability in routine practice, although inherent biases such as treatment by indication and baseline imbalances limit definitive conclusions [20,21]. Together, these findings suggest that aliskiren may be a reasonable alternative in select patients but does not displace ACEi or ARBs as guideline-preferred therapies.

This synthesis highlights potential subgroup-specific benefits of renin inhibition. In obese hypertensive men, aliskiren not only reduced mean arterial pressure but also uniquely lowered albuminuria and filtration fraction [16], suggesting that obesity-related RAAS activation may confer a favorable response profile. Similarly, trials in diabetes demonstrated that ARBs were more effective in reducing albuminuria among patients with high-normal levels [14], whereas aliskiren achieved greater relative reductions in overt microalbuminuria (−67.6% vs. −49.1% [15]). These findings indicate that the stage of renal involvement may shape therapeutic efficacy, raising the possibility of a precision medicine approach where RAAS blockade is tailored to disease phenotype rather than applied uniformly.

The trials included reveal mechanistic divergences between aliskiren and ACEi/ARBs. Aliskiren reduced filtration fraction, a hemodynamic marker linked to renal protection, whereas ACEi did not achieve this effect [16]. Conversely, ARBs significantly decreased intrarenal angiotensinogen excretion [14], a surrogate of intrarenal RAAS activation, whereas aliskiren did not. These contrasting effects suggest that renin inhibition and downstream RAAS blockade may exert complementary mechanisms rather than redundant ones, which could explain variable outcomes across different patient populations. Such mechanistic nuances underscore the importance of moving beyond BP-centric outcomes and exploring renal hemodynamics, biomarkers, and disease-stage-specific responses in future trials.

Clinically, the current evidence does not justify replacing ACEi or ARBs with aliskiren as first-line therapy, given their robust outcome data and guideline support. However, aliskiren may represent a reasonable alternative for patients intolerant to ACEi/ARB or for targeted subgroups such as obese hypertensives or those with advanced microalbuminuria. Future research should focus on large-scale, long-term RCTs powered for hard outcomes such as cardiovascular mortality, heart failure hospitalization, and end-stage renal disease. There is also a compelling need for trials in obesity-driven hypertension, where hemodynamic effects of aliskiren may be uniquely advantageous. Finally, biomarker-guided therapy using albuminuria or intrarenal angiotensinogen levels may help identify patients most likely to benefit from renin inhibition, advancing the field toward precision RAAS blockade [22,23].

This review is limited by heterogeneity in study populations, which included patients with CKD, T2DM, and obesity, thereby restricting generalizability. Several RCTs were small (N=15-237) and of relatively short duration, limiting the ability to assess long-term outcomes. Additionally, one included study was an observational registry [17], introducing bias from non-randomized treatment allocation and baseline imbalances. Finally, due to methodological differences and outcome heterogeneity, meta-analysis was not feasible, and findings should be interpreted qualitatively. These limitations highlight the need for rigorously designed, adequately powered trials before definitive clinical recommendations can be made.

## Conclusions

This systematic review demonstrates that direct renin inhibition with aliskiren provides BP-lowering efficacy comparable to ACEi and ARBs, with potential additional benefits in select subgroups such as patients with obesity-related hypertension and those with advanced microalbuminuria. However, the evidence base remains limited by small, short-term trials and the absence of robust data on hard cardiovascular and renal outcomes. While aliskiren may serve as an alternative for patients intolerant of conventional RAAS blockade, current data do not support its replacement of ACEi or ARBs as first-line therapy. Future large-scale, long-term randomized trials, incorporating biomarker-guided strategies, are essential to determine whether renin inhibition can carve a defined role in precision hypertension management.
